# An Evaluation of the Evidence on Mobile Health Applications for Mental Health, Substance Use and Delinquency in Justice-Involved Adults and Youth: a Scoping Review

**DOI:** 10.1007/s10802-025-01360-8

**Published:** 2025-10-06

**Authors:** Elyan J. J. Aarts, Bruno Verschuere, Arnold van Emmerik, Eva Billen

**Affiliations:** Department of Clinical Psychology, University of Amsterdam, Amsterdam, The Netherlands

**Keywords:** Mobile health applications, Justice-involved adults, Justice-involved youth, Quality assessment, Implementation outcomes, Effectiveness

## Abstract

Justice-involved individuals often struggle to access adequate mental healthcare, highlighting the need for new therapeutic approaches. Mobile health (mHealth) applications offer a promising solution, but research on their use in this population is limited. This scoping review maps the current literature on mobile applications targeting mental health and substance use, and delinquent behavior in justice-involved and at-risk individuals. We included full-length empirical studies and conducted descriptive quantitative analyses to summarize study characteristics. In addition, we assessed the reporting quality of each study using the CONSORT-EHEALTH checklist. Finally, we examined implementation outcomes and effectiveness through a basic qualitative content analysis. Twenty-two records, covering eighteen unique applications, were included. Most studies focused on adults in ambulant settings. This adult-centered focus overlooks the distinct developmental needs of justice-involved youth, a population that remains largely underrepresented in mHealth research. While reporting quality generally met academic standards, many studies lacked mHealth-specific details, such as user engagement metrics and digital divide demographics. Preliminary findings suggest potential benefits, including behavioral improvements, enhanced patient-clinician communication, and increased insight for the patient. However, issues such as reliance on language, privacy concerns, and technical difficulties limit usability. Reported effectiveness was often short-term or showed no significant differences between groups, possibly due to small sample sizes, high attrition, and reliance on blended treatment formats. In conclusion, mobile applications show promise for forensic mental healthcare, but further methodologically robust research is needed to improve their real-world implementation.

**Supplementary Information:**

The online version contains supplementary material available at 10.1007/s10802-025-01360-8.

In 2019, an estimated 970 million people worldwide were affected by mental health disorders, according to a report by the World Health Organization (WHO, 2022). Among them, approximately one in seven youth aged 10 to 19 years old, live with a mental disorder (WHO, 2024). Despite this high prevalence, a large proportion of treatment-seeking individuals in Western countries struggle to have their mental healthcare needs met (Alonso et al., 2004; Coombs et al., 2021; Health and Social Care Information Centre, 2014; Lennox, 2014; Prins et al., 2011; WHO, 2024).

This issue is even more pronounced among socially, psychologically, or psychiatrically vulnerable groups, such as justice-involved individuals (Nijman et al., 2017; Tomlin et al., 2020), a term used to describe anyone who has interacted with the criminal justice system as a defendant (Smith, 2022). Individuals with severe mental health disorders who have committed a crime are the primary focus of forensic psychiatry (Askola et al., 2016). The presence of severe mental illness, increased vulnerability to stigma, and a wide variation in offenses and socio-economic backgrounds (Greenberg et al., 2007; Resnick & Appelbaum, 2019) further complicates the development of effective interventions and assessment in this population. Moreover, care is often involuntary (Drieschner & Boomsma, 2008), and many patients lack insight into their own emotions and behaviors (Greenberg et al., 2007; Tervoort, 2011), making therapeutic engagement difficult.

Among them, justice-involved youth face unique and developmentally specific challenges. They often struggle with comorbid disorders, trauma, substance use, and disrupted family environments (Underwood & Washington, 2016). Therefore, effective mental health interventions for justice-involved youth require approaches that reflect their developmental needs, such as age-appropriate activities and individualised treatment approaches (Pazaratz, 2003).

These challenges highlight the need for innovative approaches beyond traditional assessment and therapeutic models, as a one-size-fits-all approach is rarely sufficient (Kip & Bouman, 2021). Recent technological advancements in mental healthcare offer promising ways to address these gaps, especially for youth who are generally more familiar with digital tools.

One notable development is the emergence of mobile health (mHealth) applications. mHealth broadly refers to mobile wireless technologies designed to improve public health, including mental health (WHO, 2018). In recent years, these applications have become widely available for patients and individuals seeking treatment in the general population (Anthes, 2016). They offer services ranging from symptom monitoring, self-management, and mindfulness training (van Emmerik et al., 2020) to exercises aimed at improving cognitive functioning (Neary & Schueller, 2018; Wang et al., 2018). Evidence suggests that mHealth applications can alleviate symptoms of mental health conditions, such as depression, anxiety, post-traumatic stress disorder (PTSD), and substance use disorders, demonstrating comparable outcomes to existing evidence-based approaches (Chandrashekar, 2018; Van Ameringen et al., 2017; Wang et al., 2018). However, information on their effectiveness among justice-involved individuals remains limited (Siezenga et al., 2022).

This gap is particularly noteworthy given the potential advantages of mHealth in forensic settings. Van Ameringen et al. (2017) outline several benefits of these applications, including their ability to provide immediate and accessible support, which can bridge the gap between therapy sessions and reduce the burden on clinicians. Additionally, applications with experience sampling methodology (ESM) (Conner et al., 2009), often allow clinicians to access real-time patient data, which can inform treatment plans, enhance patient engagement, and reduce recall bias (Potosky, 2019). This is particularly relevant for justice-involved individuals, where accurate self-reporting is often challenging (Keulen-de-Vos et al., 2011). For youth in particular, mHealth is a promising tool to deliver age-appropriate treatment, and may offer the opportunity to integrate positive peer interactions (Pazaratz, 2003). Technology also offers the possibility for caregivers, probation officers, and other adults to be involved in the child’s treatment progress. Furthermore, some mHealth applications offer customizable features, allowing for tailored interventions that better meet individual client needs (Alqahtani & Orji, 2020).

Despite these benefits, several studies have identified unique challenges faced by the forensic field during the implementation of mHealth applications. Limited access to mobile phones in high-security environments presents a major barrier, and even when available, adherence rates for mobile interventions can be low (Payne et al., 2015; Ross, 2018). Additionally, reliance on text-based applications can limit usability for individuals with low literacy or learning disabilities (Grigorenko, 2006; Kip & Bouman, 2020; Snow & Powell, 2012).

Before mHealth applications can be effectively used in forensic mental healthcare, research is needed to assess key implementation outcomes. These outcomes measure how well new treatments or services are introduced and whether they lead to successful use in practice (Proctor et al., 2011). They also help predict broader effects, such as service quality and user satisfaction. One key implementation outcome is usability, which refers to how easily and efficiently users can navigate an application to achieve their goals (International Organization for Standardization, 2013). If an application is difficult to navigate or lacks accessibility, its overall impact may be limited. By assessing implementation outcomes, researchers can identify the strengths and limitations of mHealth applications, ensuring they provide meaningful clinical benefits.

Effectiveness refers to the degree to which an intervention produces beneficial effects under “real world” circumstances (Godwin et al., 2003). Whilst closely related to the implementation outcomes described above, it is a distinct measure of how well an application contributes to a user’s treatment. Even if an application is usable and satisfactory to the user, it needs to demonstrate measurable improvements in patient outcomes to be considered successful. Whether current mHealth applications within forensic psychology are effective is still unknown (Siezenga et al., 2022) as studies on the topic are limited.

Assessing the quality of mobile health studies is complex. Multiple factors unique to mHealth, such as user engagement and an individual’s familiarity with technology, may influence outcomes. Standard reporting guidelines do not fully capture these nuances, making it difficult to evaluate the effectiveness of mHealth applications. To address this gap, Eysenbach and CONSORT-EHEALTH group (2011) developed the CONSORT-EHEALTH checklist, which expands upon existing reporting standards by incorporating mHealth specific criteria. These additional elements help establish a more reliable foundation for evaluating mobile application studies.

Currently, the evidence on mobile applications for justice-involved youth and adults is scarce. One review (Siezenga et al., 2022) has mapped applications for risk assessment, rehabilitation and reintegration of individuals who have committed offenses, however, they did not include applications designed for mental health issues or substance use. Furthermore, a report by Kip and Bouman (2020), provides examples of mobile applications available in forensic psychiatry in the Netherlands, however, they did not evaluate these applications. This underscores a significant gap in the existing research.

This scoping review aims to address this gap by charting the literature on mHealth applications specifically dedicated to the assessment, treatment, and prevention of mental health issues, substance use or delinquent behavior in the justice-involved youth or adult population, or in individuals at risk for committing a crime. Specifically, we will look at application and study characteristics such as population, recruitment setting, and application function. We also aim to critically evaluate the reporting quality of the existing research, to identify gaps in the literature that may guide future studies. Lastly, we assess the reported implementation outcomes and effectiveness of mHealth applications encountered in the literature. This approach will improve our understanding of existing applications for justice-involved individuals and support future research on the development and evaluation of more effective mobile applications tailored to their unique needs.

## Methods

The current scoping review was conducted in accordance with the PRISMA Extension for Scoping Reviews (Tricco et al., 2018).

### Protocol and Preregistration

To facilitate a broad and exploratory mapping of research on smartphone applications in forensic populations, a scoping review was considered more appropriate than a systematic review. Unlike systematic reviews, which focus on synthesizing findings from studies with comparable methodologies and outcomes, a scoping review allows for the inclusion of diverse designs and outcome measures. This approach accommodates the heterogeneity of the literature and allows for a comprehensive overview of app characteristics, research quality, implementation outcomes, and effectiveness.

A protocol was drafted according to the Preferred Reporting for Systematic Reviews and Meta-Analyses Protocols (PRISMA-P; Moher et al., 2015) and preregistered with the Open Science Framework on the 12th of February 2024 (https://osf.io/hp643/?view_only=66cd4836c8a442589c471d5b386341aa)*.*

Several minor changes were made following the pre-registration. The review initially aimed to include apps only. However, the scope was expanded to include all mHealth applications that leverage mobile devices, such as automated phone calls. Furthermore, research question three was removed due to insufficient evidence for an analysis of moderating factors, which would have been more suitable for a systematic review. The question read as follows: Which moderating factors play a role in the usability and acceptability of these smartphone apps as indicated by users or clinicians?

### Study Identification

Papers were included if they met the following criteria: (1) full-length article (i.e. no conference abstracts) written in a language mastered by the research team (Dutch, English, French, Spanish); (2) empirical study design (i.e. no reviews or study protocols); (3) focus on a mobile phone application aimed at assessment, prevention or treatment; (4) explicit assessment of mental health and substance use or delinquent behavior; (5) includes justice-involved or at risk groups (at risk of committing criminal behavior) in at least a portion of the sample. Studies with at-risk individuals were added to include prevention studies of, for example, individuals with sexual interest in prepubescent and early pubescent children (Schuler et al., 2021) or youth who are less likely to transition successfully into adulthood and/or who displayed disruptive behavior (Lau et al., 2022). No limit was established for publication year or participant age.

To identify relevant papers, we conducted a search on the 7th of December 2023 (updated on the 21st of January 2025) with search strategies developed through team discussion and with the help of an experienced librarian. The search strings and an overview of all searched databases can be found in the Supplementary materials. Additionally, we identified gray literature through a request for papers on LinkedIn, Techwijzertz.nl, a report by Kip and Bouman (2020), and by investigating the reference lists of works identified during the search.

### Study Selection

The search results from different databases were combined and de-duplicated with reference software Zotero (Corporation for Digital Scholarschip, 2023), and screened with Rayyan (Ouzzani et al., 2016). After a pilot screening trial, a list of final screener instructions was developed to ensure the reliability of independent reviewer decisions. The title and abstract of each citation were screened twice against the eligibility criteria by two independent reviewers (EA, DD), after which conflicting and uncertain screening decisions were resolved by an additional reviewer (EB).

### Data Extraction and Synthesis

A standardized coding sheet and coder instructions form (https://osf.io/9fc47*)* were developed to extract relevant information from the full-text papers and was implemented in Qualtrics (Qualtrics, 2023, Provo, UT). We extracted the following information from the included articles. (1) Study and application characteristics: We extracted information on the study population, app name, recruitment setting, target issue, application function, and study outcomes. (2) Quality assessment: The quality assessment of the reviewed reports was based on the CONSORT-EHEALTH checklist (Eysenbach & Group, CONSORT-EHEALTH group, 2011), a checklist designed to assess whether all necessary information is included when reporting mHealth studies. For the purpose of this scoping review, the checklist was shortened by combining similar items, resulting in a total of 17 items (see Supplementary materials). For example, we combined the items related to RCTs into: “If RCT: method of randomization, concealment, blinding.” Each item was scored on a 0 to 2 scale (0 = not mentioned, 1 = partially mentioned, 2 = fully mentioned, N/A = not applicable). A few additional criteria were added to assess the quality of implementation outcome studies (Maramba et al., 2019; Zapata et al., 2015). These criteria were used to assess whether studies used multiple types of methods (yes/no), which methods, and whether they were validated (yes/no). Additionally, we coded the journal impact factor.

(3) Implementation outcomes were deductively identified according to Chan and Honey’s framework (2022). Their framework includes six criteria, which can be used to evaluate the consumer perceptions of mHealth apps, namely, helpfulness, suggestions for improvements, technical issues, perceived issues, ease of use, and satisfaction. They specifically refer to usability (as defined in the introduction, see above) and acceptability. Acceptability refers to the extent to which user groups are willing to adopt the application for the tasks it is designed to support (Dillon, 2001, p.2). Of note, there is overlap with application engagement, which captures how actively and meaningfully users interact with an application, including affective, cognitive, and behavioral dimensions (Kelders et al., 2020). An example of behavioral engagement is frequency of use and adherence, while cognitive engagement relates more to how individuals think about the application, and affective engagement concerns how they feel when using the application. (4) Effectiveness factors were identified through an inductive approach.

Data analysis involved quantifying results and calculating the frequency of extracted items, as suggested by Pollock et al. (2023). Furthermore, a basic qualitative content analysis was used to examine implementation outcomes and effectiveness. For implementation outcomes, findings from the studies were categorized using six predefined themes from a previous meta-analysis (Chan & Honey, 2022). While sorting findings into these categories, additional subcategories emerged. For effectiveness, findings were summarized rather than categorized. If a study reported effect sizes, these were based on Cohen’s d (Sense-It: Ter Harmsel et al., 2023a), regression coefficients, and odds ratios (Phone Coach system RealVicotry Program; Burraston et al., 2014).

## Results

**Fig. 1:**
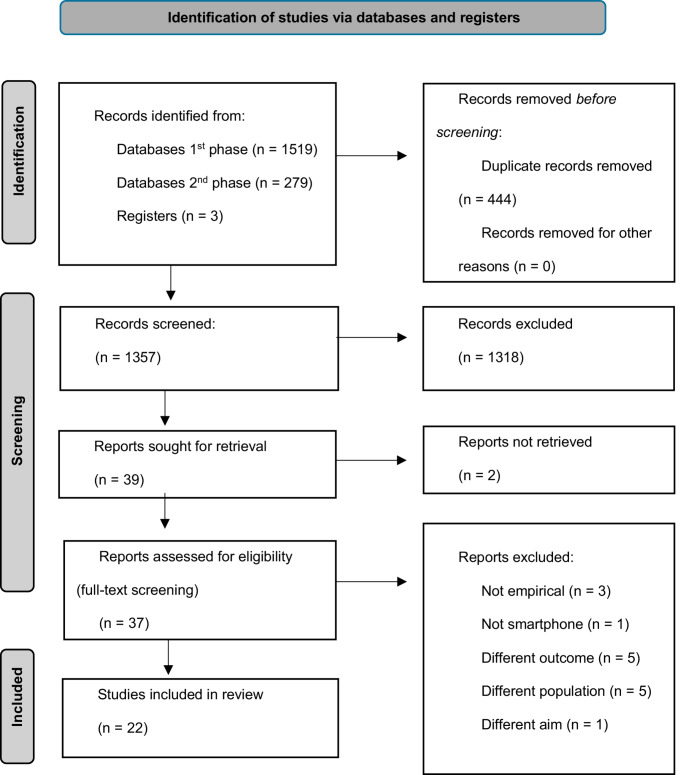
PRISMA flow-chart

The initial and updated search combined identified 1801 records. Following the title and abstract screening, the independent reviewers (EA, DD) reached a 96% agreement on the included articles. A third independent reviewer (EB) assessed and screened the remaining 4%. After the title and abstract screening, 1318 records were excluded. Most were excluded because they were unrelated to the topic of this review, for example, court case descriptions and animal studies. Other studies were excluded for the following reasons: narrative/non-empirical review or essay (*n* = 119), not full-text (e.g. conference abstract or study protocol) (*n* = 19) or unfamiliar language (*n* = 2). A total of 139 articles addressed smartphone applications, of which 100 were excluded because they were not developed or tested within forensic groups (e.g., police officers, victims, nurses) or the outcome differed from the goal of this scoping review (e.g., sexual health, employment, or disease prevention). Thirty-nine records were retrieved for full-text screening. Two records could not be retrieved during the initial search. However, an article on the same application was identified and included during the update (McCrady et al., 2025). During the full-text screening, records were excluded because they covered a protocol or review (*n* = 3), or the application did not match all the inclusion criteria (e.g. focus on reintegration instead of mental health and substance use/delinquent behavior) (*n* = 12). Please refer to Fig. 1 for a summary of the identification, screening and inclusion process.

In the end, twenty-two records were included, covering eighteen unique applications (see Table 1 for a full overview of included studies). Most of these studies were conducted in Europe (41%) or North America (41%). The publication years ranged from 2012 to 2023.

**Table 1:** Overview of included studies

Authors & year	Mobile application name	Location
Hulsmans et al., 2023^[1]^	No Name: “Daily Monitoring”	Europe, Netherlands
Carswell et al., 2022^[2]^	Continuing Care	North America, USA
Horst et al., 2023^[3]^	HKT-R Spider	Europe, Netherlands
Buitenweg et al., 2019^[4]^	QoL-ME	Europe, Netherlands
McGreevy, 2017^[5]^	Changing Lives	Europe, UK/Northern Ireland
Johnson et al., 2016^[6]^	A-CHESS^6^	^1^ North America, USA
Ross et al., 2022^[7]^	e-recovery^7^ (based on A-CHESS)	^2^Australia
Ter Harmsel et al., 2023a^[8]^ Ter Harmsel et al., 2023b^[9]^ Ter Harmsel et al., 2021^[10]^	Sense-it biocueing^8,9,10^	Europe, Netherlands
Sugie, 2018^[11]^	No Name: “Disadvantaged Groups”	North America, USA
Langdon et al., 2022^[12]^	No Name: “Continued Engagement”	North America, USA
Perdacher et al., 2022^[13]^ Perdacher et al., 2024^[14]^	Stay Strong^13,14^	Australia
Schuler et al., 2021^[15]^	Troubled Desire	Europe, Germany
Lau et al., 2022^[16]^	No name: “New Generation”	Asia, Hong Kong
Sugie & Augustine, 2020^[17]^	No Name: “Social Support”	North America, USA
Cox, 2016^[18]^	Look	North America, USA
Burraston et al., 2014^[19]^ Burraston et al., 2012^[20]^	Phone Coach System RealVicotry Program^19,20^	North America, USA
Leijse et al., 2024^[21]^	Feelee	Europe, Netherlands
McCrady et al., 2025^[22]^	B-SMART	North America, USA

Note. Superscripted ^[numbers]^ are used to cross-reference papers across Table

### App and Study Characteristics

Among the eighteen applications identified (from twenty-two papers), some included multiple populations, settings, or behaviors, resulting in reported percentages that do not to sum up to 100%. Most applications were tested in justice-involved adults (77%), followed by justice-involved youth (17%), at-risk youth (11%), and at-risk adults (6%). Refer to Table 2 for an overview of the average age and gender distributions. Participants were most often recruited from ambulant (39%) or probation settings (28%). Most apps were aimed at treatment (67%) and/or assessment (39%) rather than prevention (17%). Additionally, the main target was mental health (44%) compared to substance use (39%) and delinquent behavior (39%). Mental health issues were broadly referenced as “symptoms”, “mood”, or “quality of life”, while delinquency apps focused on “criminal thinking and behavior”, “deviant sexual desire” or “(re-)offending”. In terms of study outcomes, most of the twenty-two papers studied usability (50%) or feasibility (41%), while only 18% reported effectiveness or app development (11%). For more details per record, please refer to Table 2.

**Table 2:** App and study characteristics

Application/Article	Population (*N*)	Age; Gender	Recruitment setting	Application function	Target issue	Study outcome
“Daily Monitoring”^[1]^	Forensic adults, forensic & at-risk youth (50)	M = 21.4 (SD = 5.1, range 14–33); 56% male	Ambulant, Juvenile detention, Residential care	Assessment, treatment	MH: internalizing and externalizing symptoms	Usability, feasibility
Continuing Care^[2]^	Forensic adults (15)	M = 49.3 (SD = 8.3); 86.7% male	Probation, parole	Prevention, treatment	SUD; Delinquency: criminal thinking & behavior	Usability, feasibility
HKT-R Spider^[3]^	Forensic adults (32)	M = 35; 80% male	Psychiatric clinic	Assessment	MH: psychosis, impulsivity; Delinquency: antisocial & hostility; Addiction	Usability, inter-rater agreement
QoL-ME^[4]^	Forensic adults (59)	M = 40.8 (SD = 15); 80% male	Ambulant, psychiatric clinic	Assessment	MH: quality of life	Usability
Changing Lives^[5]^	Forensic adults (Unclear)	N/A	Probation	Inform	MH: mood, stress, symptoms; addiction	N/A
A-Chess^[6]^	Forensic adults (30)	52% = 18–24; 87% male	Outpatient drug program	Prevention, treatment	Substance abuse	Feasibility
eRecovery^[7]^	Forensic adults (36)	M = 36 (range = 24–56); 58% male	Ambulant, community service	Prevention, treatment	Substance abuse	Usability, feasibility
Sense-It Biocueing^[8], [9], [10]^	Forensic adults (21^[8]^, 25^[9]^, 10^[10]^)	M = 29.88 (SD = 10.51)/ M = 34.9 (SD = 13.29); 90–92% male	Ambulant	Treatment	Delinquency: aggression	Usability, feasibility, effectiveness
“Disadvantaged Groups”^[11]^	Forensic adults (135 + 21)	> 18; 100% male	Former inmates	Assessment	MH: emotional well-being	Usability
“Continued Engagement”^[12]^	Forensic adults (8)	M = 47.4 (SD = 11.29); 87.5% male	Ambulant, probation	Treatment	Opioid abuse	Usability, feasibility
Stay Strong^[13, 14]^	Forensic adults (27^[13]^, 82^[14]^)	> 18; 70%^[13]^ female/60.6% female^[14]^	Prison	Treatment	MH: psychological symptoms; substance misuse	Feasibility^[13]^, effectiveness^[14]^
Troubled Desire^[15]^	Forensic adults, at-risk adults (7496)	80% was < 40 years; 90.9% male	General population	Assessment, treatment	Delinquency: deviant sexual desires	Characteristics of app users
“New Generation”^[16]^	At-risk youth (591)	N/A	General population, correctional services	Treatment	Delinquency: offending, drug use, gambling, drinking	Feasibility
“Social Support”^[17]^	Forensic adults (135)	> 18; 100% male	Parole	Assessment	MH: general well-being	ESM results
Look^[18]^	Forensic adults (44)	> 18; 100% male	Ambulant	Assessment	Delinquency: deviant sexual desires	Validity
Phone Coach System RealVicotry Program^[19], [20]^	Forensic youth (39 treatment, 31 control)	M = 16.07 (SD = 1.21); 89% male	Probation, juvenile court	Treatment, Prevention	Delinquency: recidivism	Effectiveness
Leijse et al., 2024^[21]^	Forensic youth (4)	M = 18.5 (SD = 0.58); 100% male	Ambulant	Treatment	MH: emotion regulation	Usability, feasibility
McCrady et al., 2025^[22]^	Forensic adults*	> 21; 77% male	Local DWI counseling and court programs	Treatment	MH: effective communication, social support; alcohol misuse	Co-design, usability

Note. *MH *Mental Health, *SUD *Substance use disorder, *M *mean, *SD *standard deviation, *N/A *information not available. The study “Disadvantaged groups” included 156 subjects of which 135 participated in the smartphone study, and 21 were interviewed for the usability. *The information on the sample from McCrady et al. (2025) only includes the sample from the usability study, not the co-design

**Fig. 2:**
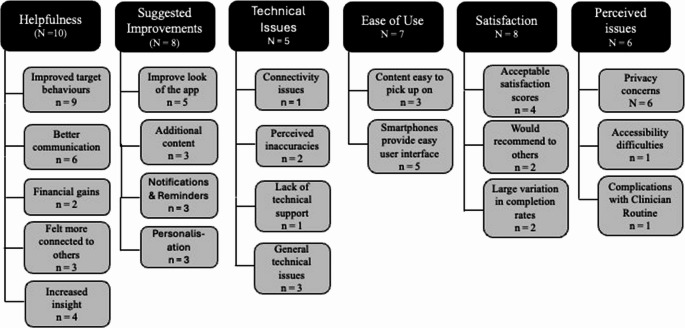
Implementation outcomes themes and sub-themes. *Note*. Figure design inspired by Faber et al. (2023). The figure shows the six themes identified by Chan and Honey (2022) in the black boxes, while the gray boxes show the related sub-themes. Not all usability studies addressed each theme; the total count for each is shown in the corresponding box

### Evaluation of Reporting Quality

The CONSORT-EHEALTH checklist (Eysenbach & CONSORT-EHEALTH group, 2011) was used to review the general quality of the mHealth reports included in this scoping review. Overall, less than 30% of checklist items were scored 1 or 0. However, Lau et al. (2022) had 53% of items scored 1 or 0, indicating that key information was often partially or not reported. It is important to note that this was a pilot study, and missing information is not uncommon during earlier testing phases. Additionally, some key details were missing, including the user metrics that were collected but not reported. The study also used a self-developed questionnaire, which had not yet been fully validated. Furthermore, there was no mention of ethical approval, nor could the authors be contacted for clarification.

In terms of section-specific completeness, most studies were remarkably thorough in the introduction (*M =* 1.95) and methods (*M* = 1.73) sections. However, item 7 (i.e. sample size justification) in the methods section had a mode of 0, meaning that most studies did not provide a justification for the reported sample size. The results section had the lowest average score (*M =* 1.55), primarily due to missing details on participant flow, digital divide demographics (i.e. demographics related the gap between individuals who are able and those who are unable to benefit from online health information and services; Jenkins et al., 2022), and user metrics. In the discussion section (*M* = 1.79), the lowest scoring item was item 14, indicating that some studies did not adequately discuss the generalizability of their results. Across all items, the lowest scoring was item 17, with a mode of 0, indicating that most studies were not (pre)registered (77%). The journal impact factor varied from lower than 2 (37%), between 2 and 4 (37%), and higher than 4 (16%). Two sources were from dissertation articles and thus were not evaluated for publication standard (for more details on scoring, please refer to the Supplementary materials) .

### Implementation Outcomes

Our next step was to assess the studies (*n* = 12) that evaluated implementation outcomes (e.g. feasibility, usability) (Buitenweg et al., 2019; Carswell et al., 2022; Horst et al., 2023; Hulsmans et al., 2023; Langdon et al., 2022; Leijse et al., 2024; McCrady et al., 2025; Perdacher et al., 2022; Ross et al., 2022; Sugie, 2018; Ter Harmsel et al., 2023a, b). Among these studies, 50% utilized a single assessment method (50% qualitative, 50% quantitative), while the remaining 50% combined qualitative methods with quantitative or heuristic approaches (e.g., logging data). Only 42% used validated instruments. However, it is important to note that qualitative studies often use study-specific interview questions, which may not undergo traditional validation processes (Creswell & Poth, 2016) (Table 3).

**Table 3:** Quality assessment of twelve implementation outcome studies

App	Type of method	Specific method	Validated method
“Daily Monitoring”^[1]^	Qualitative Quantitative	Structured interview Scale from 1 to 5	No
Continuing Care^[2]^	Quantitative	Feasibility questionnaire scale from 1 to 5	No
HKT-R Spider^[3]^	Qualitative	One open question	No
QoL-ME^[4]^	Quantitative	System Usability Scale (SUS; Brooke, 1996)	Yes
e-Recovery^[7]^	Qualitative Heuristic	Semi-structured interview Duration/frequency of use, daily check-ins	No Yes
Sense-it^[8]^	Quantitative Qualitative	SUS Semi-structured interview	Yes No
Sense-it^[9]^	Quantitative Qualitative	SUS Semi-structured interview	Yes No
“Disadvantaged Groups”^[11]^	Qualitative Heuristic	Semi-structured interview Location estimates, attrition rates	No No
“Continued Engagement”^[12]^	Qualitative	Semi-structured interview	No
Stay Strong^[13]^	Qualitative	Semi-structured interview	No
Feelee^[21]^	Qualitative, Heuristic	Semi-structured interview, app usage	No
B-SMART^[22]^	Quantitative	SUS	Yes

Note. Quality assessment based on Maramba et al. (2019) and Zapata et al. (2015)

The results of the qualitative content analysis were based on the six themes identified and defined by Chan and Honey (2022), namely, helpfulness, suggested improvements, technical issues, ease of use, satisfaction and perceived issues. Within these themes, several sub-themes emerged during the analysis. Figure 2 provides a complete overview of all the (sub)themes and the frequency with which they were reported. Not all twelve studies addressed each theme.

The most commonly discussed theme was “helpfulness” (10/12), which refers to the perceived positive impact the application has on symptom management, mental healthcare, and skill learning. In nine studies, participants explicitly reported that the application helped them to improve their target behaviors (e.g. reduce substance use, develop coping strategies). Six studies mentioned that using the application helped to open the conversation and build rapport with the clinician during the session.

“Suggested improvements” and “satisfaction” were mentioned by 8 out of 12 studies. “Suggested improvements” refers to user suggestions to improve the application. Examples are changes to the look or design of the application (e.g. more contrast) (5/8), or a desire for more (or less) reminders (3/8). “Satisfaction” covers the general satisfaction of users while using the application. In 4 out of 8 studies, satisfaction was measured with the system usability scale (SUS; Brooke, 1996) and resulted in acceptable scores.

“Ease of use” was mentioned by 7 out of 12 studies and refers to the user-friendliness of the application. Five out of 7 studies mentioned that a smartphone provided an easy user interface that most individuals can work with.

The least frequently covered themes were “perceived issues” (6/12) and “technical issues” (5/12). “Perceived issues” pertains to perceived barriers to using the application. The main barrier (6/6) was privacy concerns, especially regarding applications that track GPS data. One study (Ross et al., 2022) highlights the heavy reliance on language as an accessibility barrier for users with low literacy levels, an issue previously highlighted by Kip et al. (2020). Besides general literacy (i.e. the ability to read and write), digital literacy (i.e. the ability to access, understand and apply digital health information; Jenkins et al., 2022) can also be a barrier. The theme “technical issues” covers issues that prevent proper use of the application or device. For example, two studies mentioned perceived inaccuracies in the application features (e.g. inaccurate step tracking).

### Effectiveness

Three articles reported effectiveness of a mobile intervention, namely, Sense-it (Ter Harmsel et al., 2023a, b), the Phone Coach System RealVicotry Program (Burraston et al., 2014), and Stay Strong (Perdacher et al., 2024). Effect sizes are reported as detailed in the original publication or were calculated based on the available statistics.

#### Sense-IT

Sense-IT is a mobile health application paired with wearable technology designed to support aggression regulation therapy by delivering real-time biofeedback based on heart-rate variability (Ter Harmsel et al., 2023a, b). It was tested in a mixed-methods study combining a pre-posttest design and a single-case experimental design (SCED) among forensic outpatients. Although results suggested a short-term reduction in self-reported aggression (Cohen’s *d* = −0.457, *p* = 0.041 L) with a small to medium effect (Fritz et al., 2012), no long-term effects were found, and no significant changes emerged in other outcomes such as therapist-reported aggression or emotion regulation. In the SCED, they found very small to moderate effects (Parker et al., 2009) for behavioral control (IRD = 0.05–0.55) and aggressive behavior (IRD = 0.05–0.51) at the individual-level. Nonetheless, the absence of group-level effects indicates the limited overall impact of the intervention.

The evaluation of the study highlights several methodological challenges, including high drop-out rates, small sample sizes, and the inability to disentangle the app’s effects from the concurrent treatment programme. Strong points include the use of multiple assessment methods (self-and other-report, ecological momentary assessment, physiological data) and within-person control through SCED design.

#### Phone Coach System RealVicotry Program

The RealVictory program combined cognitive-behavioral rehabilitation with year-long automated phone calls, aiming to reduce recidivism in youth with delinquent behavior (Burraston et al., 2014). Participants could personalize the content and frequency of calls, which included supportive pre-recorded messages from family and friends. Participants who consistently engaged with both the training and phone calls were significantly less likely to be rearrested than participants in the standard treatment (OR = 0.21), indicating a large protective effect (Chen et al., 2010). They also had a lower total number of rearrests (IRR = 0.43), reflecting a 57% reduction compared to standard treatment. Cognitive- behavioral training alone was also associated with a 45% reduction in the total number of rearrests (IRR = 0.55), while participants with low engagement with the phone calls did not improve more than the standard treatment group.

The evaluation of the study highlights several methodological challenges, including small sample sizes and post-hoc grouping (no randomization), which raises concerns about bias. Nonetheless, the study did include a separate control group and show low drop-out rates.

#### Stay Strong

Stay Strong is a mobile app designed by and for Aboriginal and Torres Strait Islander people in prison, focusing on well-being, substance use, and reintegration support (Perdacher et al., 2024). In a pilot randomized control trial across three high-security prisons, participants were randomized to receive the intervention immediately or after a delay. No significant differences were found between groups, but all participants showed improvements over time in empowerment (Cohen’s *d* = 0.99), well-being (Cohen’s *d* = 0.76, and distress (immediate group Cohen’s *d* = 0.32, and delayed group Cohen’s *d* = 0.49). These correspond to large, medium-to-large, and small-to-medium effects, respectively (Fritz et al., 2012). Despite the general improvements, the lack of significant group effects suggests that Stay Strong did not provide additional benefits beyond existing Indigenous Mental Health Intervention Program services.

The evaluation of the study highlights several methodological challenges. There were high attrition rate in both groups. It was unclear whether improvements are caused by the app or general factors (e.g., receiving mental health services).

## Discussion

This scoping review evaluated the current literature on mobile health (mHealth) applications for the assessment, treatment, and prevention of mental health and substance use problems, as well as delinquent behavior, in justice-involved youth and adults. Only eighteen unique applications met our criteria, underscoring that adoption in forensic settings is still at an early stage despite widespread availability of consumer health apps.

Most evaluated apps targeted substance-use treatment for adults, with little attention to other clinically relevant domains such as trauma and self-regulation. Although the emphasis on substance use is understandable given its high prevalence in justice-involved populations (Marquant et al., 2021), the narrow scope suggests that mHealth tools may not yet fully address the complex needs of forensic populations at large.

Equally important, this adult-centered focus overlooks the distinct developmental needs of justice-involved youth, a population that remains largely underrepresented in mHealth research. This review identified only four applications developed specifically for youth, compared to fourteen targeting adults. Encouragingly, a surge in recent protocols indicates a growing interest in youth-specific research. The development of applications such as the Teen Empowerment through Computerized Health (Helseth et al., 2022) and BrotherlyACT (Emezue et al., 2023) shows that mHealth tools may soon be used to address substance use and delinquency problems in justice-involved youth.

Beyond youth-only applications, digital tools can also enlist their social environment. Schaeffer et al. (2022) developed a caregiver-focused app that allowed parents to set expectations and monitor the behavior of adolescents with conduct problems. Designing paired modules for youth, caregivers, probation officers or other supportive adults, could stimulate personal change (Jerrott et al., 2022) and enhance treatment engagement (Tolou-Shams et al., 2019). However, privacy concerns play a large role when information is shared with anyone other than the youth themselves. Clear boundaries and transparency regarding private information needs to be established in advance (Tolou-Shams et al., 2019).

Overall, most applications were well-received by users and showed some promise in improving behavioral outcomes and communication. However, barriers such as low (digital) literacy, privavy concerns, and technical issues remain an issue. Evidence of effectiveness remains methodologically weak due to small sample sizes and high attrition rates, or are limited to short-term or inconsistent effects. In addition, reporting standards were generally strong, but gaps remain in reporting of user metrics, digital divide characteristics, sample size justification, and conducting preregistration. These challenges point to the need for stronger methodological rigor and implementation planning in future mHealth research.

The gap between the potential benefits of mHealth and its current use in practice remains large. High hardware costs, staff-training needs and interoperability with existing technologies (e.g. many different platforms) are potential implementation obstacles (Kip et al., 2021; Kip et al., 2021). Kip et al. (2025) propose a comprehensive roadmap that treats development, implementation and evaluation as an iterative process. The process doesn’t end after every step, but it’s a constant cycle of criticizing and adapting your work and it’s practical use. It’s important to plan for real-world integration from day one, for example, by building on platforms that are already in use. Furthermore, involving stakeholders in the design process through co-creation, ensures that the end-product aligns with their needs and desires.

This review has several notable strengths. It is the first to provide a comprehensive overview of mHealth applications targeting mental health, substance use and delinquency in justice-involved individuals. We aimed to capture a broad range of relevant literature by expanding our search beyond conventional healthcare databases by including Web of Science and SocINDEX. Furthermore, all titles and abstracts were screened twice by independent reviewers.

However, several limitations should be acknowledged. First, our ability to draw conclusions may be limited by the small number of included studies and language restrictions (English, Dutch, Spanish, and French). Second, the heterogeneity in study designs, sample sizes, and outcome measures limited the possibilities for aggregation of the data and direct comparisons between the included studies. Nonetheless, mapping these varied studies provides a starting point for better understanding the role of mHealth applications in forensic settings. In a similar vain, the heterogeneity of populations (adults, youth, community/outpatient, restricted/inpatient/prison) limits the generalizability between groups. The majority of the applications were tested in adult populations, and their findings may not apply to youth. Historically, many jurisdictions have placed justice-involved youth in programmes modelled on adult services (Shelton, 2005), despite evidence that developmentally tailored content is more effective. However, these existing applications may still serve as valuable starting points, provided they are restructured to align with the unique developmental, social, and legal contexts of young individuals. Furthermore, implementation outcomes should be considered per setting. In community contexts most participants possess a personal smartphone, whereas high-security facilities prohibit them outright, or restrict access to staff-controlled applications, adding an extra layer of privacy concerns. Lastly, while articles were initially screened twice by independent reviewers, data extraction was performed by a single researcher, introducing a potential risk of bias. To mitigate this risk, we used a pre-designed and thoroughly reviewed coding sheet to guide extraction. Additionally, preregistration enhanced transparency by ensuring minimal post-hoc adjustments.

The findings of this scoping review highlight several important areas for future research. First, developments of mobile applications targeting the needs of justice-involved youth are urgently needed. Additionally, it’s highly recommended to broaden the scope of applications to include a range of issues, such as self-regulation and trauma. Second, larger controlled studies with long-term follow-up are required to accurately establish the impact of the existing tools. One of the main issues contributing to low sample sizes is high attrition rates. Various efforts can be made to mitigate this. For example, user engagement can be improved by involving end-users in the design of the application, and by incorporating clinician or peer support features (Torous et al., 2018). Additionally, addressing accessibility barriers and technical issues, and improving privacy protections are necessary to improve implementation outcomes.

Lastly, more rigorous practices are needed to ensure the reliability and transparency of findings. Researchers should pre-register study protocols on open science platforms to minimize bias and enhance the credibility of their results (Nosek et al., 2018). Furthermore, standardized and validated approaches should be adopted to improve reliability and facilitate meaningful cross-study comparisons. Furthermore, reporting guidelines should be adopted that extend beyond the standard academic criteria by incorporating user metrics and digital divide demographics (Eysenbach & CONSORT-EHEALTH group, 2011).

## Conclusion

In conclusion, while mHealth applications show great potential for forensic mental healthcare, more methodologically sound research is needed (especially for youth!) to optimize their effectiveness and ensure their benefits are fully realized. Although only a limited number of studies were identified in this review, we expect to see a growing body of research and development in the coming years, as interest in mHealth applications continues to increase.

## Supplementary Information

Below is the link to the electronic supplementary material.

ESM 1 (DOCX 65.0 KB)

## Data Availability

Materials can be accesses on OSF: https://osf.io/9fc47.
