# Supplementary material for: An Evaluation of the Evidence on Mobile Health Applications for Mental Health, Substance Use and Delinquency in Justice-Involved Adults and Youth: a Scoping Review
Source: Res Child Adolesc Psychopathol. 2025 Oct 6;53(12):1829–42. doi: 10.1007/s10802-025-01360-8 (PMC12718316; doi:10.1007/s10802-025-01360-8)
Supplement: Supplementary file 1 — (DOCX 65.0 KB) [file 10802_2025_1360_MOESM1_ESM.docx]

Supplementary Material: Search Strategies & Reporting Quality Assessment

**Appendix A: Search Strategies**

The search below yielded 1.519 results on 7 December, 2023; PsycInfo (386 results), Medline (464 results), Web of Science Core Collection (407 results) and SocINDEX (262 results).

**PsycInfo (Ovid, APA PsycInfo, 1806 to Month Week 4 2023)**

1. mobile applications/ OR mobile assessment/ OR mobile phones/ OR (app OR apps OR  iphone* OR  mobile app* OR mobile assessment* OR mobile device* OR mobile phone* OR smart phone* OR smartphone* OR smart watch OR smartwatch OR wearable app*).ti,ab,id.
2. correctional institutions/ OR correctional psychology/ OR crime/ OR criminal behavior/ OR criminal offenders/ OR forensic psychiatry/ OR formerly incarcerated/ OR incarcerated/ OR incarceration/ OR juvenile delinquency/ OR juvenile justice/ OR mentally ill offenders/ OR prisons/ OR (behind bars OR conditional release* OR convicts OR correctional OR crime* OR criminal* OR delinquen* OR detain* OR detent* OR forensic OR imprison* OR incarcerat* OR inmate* OR jail* OR offender* OR offence* OR parole* OR penitentiar* OR perpetrator* OR prison* OR remand cent* OR secure facilit*).ti,ab,id.
3. 1 AND 2

Key: / = subject heading, ti = title, ab = abstract, id = key concepts (other keywords added by PsycInfo indexers to supplement the subject headings), ADJn = word distance of maximum n words, * = unlimited number of characters

**MEDLINE (Ovid MEDLINE ALL, including Epub Ahead of Print, In-Process, In-Data-Review & Other Non-Indexed Citations and Daily, 1946 to December 06, 2023)**

1. mobile applications/ OR smartphone/ OR cell phone/ OR (app OR apps OR  iphone* OR  mobile app* OR mobile assessment* OR mobile device* OR mobile phone* OR smart phone* OR smartphone* OR smart watch OR smartwatch OR wearable app*).ti,ab,kf.
2. correctional facilities/ OR jails/ OR crime/ OR criminal behavior/ OR criminals/ OR juvenile delinquency/ OR prisons/ OR prisoners/ OR forensic psychiatry/ OR  (behind bars OR conditional release* OR convicts OR correctional OR crime* OR criminal* OR delinquen* OR detain* OR detent* OR forensic OR imprison* OR incarcerat* OR inmate* OR jail* OR offender* OR offence* OR parole* OR penitentiar* OR perpetrator* OR prison* OR remand cent* OR secure facilit*).ti,ab,kf.
3. 1 AND 2

Key: / = medical subject heading (MeSH), ti = title, ab = abstract, kf = author supplied keywords, ADJn = word distance of maximum n words, * = unlimited number of characters

**Web of Science Core Collection (Web of Science Core Collection Editions: Science Citation Index Expanded (SCI-EXPANDED), 1975 - present, Social Sciences Citation Index (SSCI), 1975 - present, Arts & Humanities Citation Index (A&HCI), 1975 - present, Emerging Sources Citation Index (ESCI), 2005 - present))**

1. TS=("app" OR "apps" OR " iphone*" OR " mobile app*" OR "mobile assessment*" OR "mobile device*" OR "mobile phone*" OR "smart phone*" OR "smartphone*" OR "smart watch" OR "smartwatch" OR "wearable app*")
2. TS=("behind bars" OR "conditional release*" OR "convicts" OR "correctional" OR "crime*" OR "criminal*" OR "delinquen*" OR "detain*" OR "detent*" OR "forensic" OR "imprison*" OR "incarcerat*" OR "inmate*" OR "jail*" OR "offender*" OR "offence*" OR "parole*" OR "penitentiar*" OR "perpetrator*" OR "prison*" OR "remand cent*" OR "secure facilit*")
3. TS=("teen*" OR "youngster*" OR "young adult*" OR "young people" OR "youth*" OR "minors*" OR "under ag*" OR "underag*" OR "juvenile*" OR "girl*" OR "boy*" OR "preadolesc*" OR "adolesc*" OR "patient*" OR "prisoner" OR "delinquent*" OR "convicts" OR "inmate*" OR "offender*" OR "criminals" OR (("forensic" OR "criminal") NEAR/2 ("population*" OR "psychiatry" OR "psycholog*" OR "mental health")) OR "perpetrator*" OR "parolee")
4. #1 AND #2 AND #3

Key: TS = topic, which includes title, abstract, author keywords and Web of Science Keywords Plus, NEAR/n = word distance of maximum n words, * = unlimited number of characters

**SocINDEX (Proquest)**

1. SU("social media mobile apps" OR “cell phones”) OR TI("app" OR "apps" OR " iphone*" OR " mobile app*" OR "mobile assessment*" OR "mobile device*" OR "mobile phone*" OR "smart phone*" OR "smartphone*" OR "smart watch" OR "smartwatch" OR "wearable app*") OR AB("app" OR "apps" OR " iphone*" OR " mobile app*" OR "mobile assessment*" OR "mobile device*" OR "mobile phone*" OR "smart phone*" OR "smartphone*" OR "smart watch" OR "smartwatch" OR "wearable app*") OR KW("app" OR "apps" OR " iphone*" OR " mobile app*" OR "mobile assessment*" OR "mobile device*" OR "mobile phone*" OR "smart phone*" OR "smartphone*" OR "smart watch" OR "smartwatch" OR "wearable app*")
2. SU("correctional" OR "crime*" OR "criminal*" OR "delinquen*" OR "detent*" OR "forensic" OR "imprison*" OR "parole*" OR "prison*") OR TI("behind bars" OR "conditional release*" OR "convicts" OR "correctional" OR "crime*" OR "criminal*" OR "delinquen*" OR "detain*" OR "detent*" OR "forensic" OR "imprison*" OR "incarcerat*" OR "inmate*" OR "jail*" OR "offender*" OR "offence*" OR "parole*" OR "penitentiar*" OR "perpetrator*" OR "prison*" OR "remand cent*" OR "secure facilit*") OR AB("behind bars" OR "conditional release*" OR "convicts" OR "correctional" OR "crime*" OR "criminal*" OR "delinquen*" OR "detain*" OR "detent*" OR "forensic" OR "imprison*" OR "incarcerat*" OR "inmate*" OR "jail*" OR "offender*" OR "offence*" OR "parole*" OR "penitentiar*" OR "perpetrator*" OR "prison*" OR "remand cent*" OR "secure facilit*") OR KW("behind bars" OR "conditional release*" OR "convicts" OR "correctional" OR "crime*" OR "criminal*" OR "delinquen*" OR "detain*" OR "detent*" OR "forensic" OR "imprison*" OR "incarcerat*" OR "inmate*" OR "jail*" OR "offender*" OR "offence*" OR "parole*" OR "penitentiar*" OR "perpetrator*" OR "prison*" OR "remand cent*" OR "secure facilit*")
3. S1 AND S2

Key: SU = Subject Headings, TI = title, AB = abstract, KW = author supplied keywords, Nn = word distance of maximum n words

**Appendix B: Reporting Quality Assessment**

The general quality assessment of the reviewed papers in “An Evaluation of the Evidence on Mobile Health Applications for Mental Health, Substance Use and Delinquency in Justice-Involved Adults and Youth: A Scoping Review” was based on the CONSORT-EHEALTH Checklist (Eysenbach & Group, 2011). Table A details all the items as they were used in the evaluation. Table B details the results per article included in the scoping review.

**Items**

For the purpose of this scoping review, the checklist was shortened by combining several items into one. In Table A we have detailed the exact wording of the items:

**Table A**

*Item Adapted from the CONSORT-EHEALTH Checklist*

| Item 1) | Does the title/abstract mention: mode of delivery, non-web-based component, the target group? (1a) |
| --- | --- |
| Item 2) | Is there a clear description of the scientific background, rationale for the application? What is the problem? What are the reasons for and what is the context for this specific study? What is the population? (2a) |
| Item 3) | Are the objectives/hypotheses (for RCTs) or aims (for all other studies) clearly mentioned? |
| Item 4) | Is there a clear description of the participants (inclusion/exclusion), setting, any e-health specific criteria, recruitment (4a) |
| Item 5) | Are there enough details about the app: platform, features, content, user flow, any theory basis. If a co-design study, do they describe the iterative design process? (5) |
| Item 6) | Primary and secondary outcomes pre-defined, how are they measured? Are measures validated? (6a) |
| Item 7) | Is there a justification for the sample size? Do they mention a target number? Was expected attrition taken into account when determining the sample size? (7a) |
| Item 8) | If RCT: method of randomization, concealment, blinding (8, 9, 11) |
| Item 9) | For quantitative studies: Stats used, handling of missing data, intention-to-treat vs. per-protocol, etc. For qualitative: analysis approach (thematic analysis, etc.) (12a) |
| Item 10) | Participant flow diagram or description (how many were approached, eligible, enrolled, dropped out, lost to follow-up) (13b), how many participants were included in the analysis and completed the intervention (16)? |
| Item 11) | Baseline demographics and clinical characteristics (especially demographics associated with digital divide issues such as age, gender, education, socio-economic background, technology literacy) (15) |
| Item 12) | Main results of primary and secondary outcomes, but also metrics of use and intensity of use (engagement rates, average session length etc) (17a) |
| Item 13) | Interpretation of results in context: restate study questions and summarize the answers suggested by the data (22) |
| Item 14) | Generalizability: do they discuss how results might generalize beyond the study setting or in other populations? (21) |
| Item 15) | Limitations: acknowledgement of limitations related to e-health (internet access, multiplicity of outcomes, potential biases due to non-use of the intervention or unexpected events) (20) |
| Item 16) | Ethics approval obtained, privacy/data security, conflict of interest? (X26, X27) |
| Item 17) | (pre-)Registration? (23) |

*Note.* The number and letter (e.g. 1a) at the end of the item description refers to the corresponding item in the full CONSORT-EHEALTH Checklist.

**Scoring**

For the purpose of evaluating the quality of the papers in the review, a scoring system was established:

N/A = not applicable to this study

0 = not mentioned

1 = partially mentioned

2 = fully mentioned

**Results**

**Table B**

*General Quality Assessment based on CONSORT-EHEALTH Checklist*

| Article | Item 1 | Item 2 | Item 3 | Item 4 | Item 5 | Item 6 | Item 7 | Item 8 | Item 9 | Item 10 | Item 11 | Item 12 | Item 13 | Item 14 | Item 15 | Item 16 | Item 17 |
| --- | --- | --- | --- | --- | --- | --- | --- | --- | --- | --- | --- | --- | --- | --- | --- | --- | --- |
| Hulsmans et al., 2023^[1]^ | 2 | 2 | 2 | 2 | 2 | 2 | 1 | N/A | 2 | 1 | 2 | 2 | 2 | 2 | 2 | 2 | 2 |
| Carswell et al., 2022^[2]^ | 2 | 2 | 2 | 2 | 2 | 2 | 1 | N/A | 1 | 2 | 2 | 2 | 2 | 2 | 1 | 2 | 0 |
| Horst et al., 2023^[3]^ | 2 | 2 | 2 | 2 | 2 | 2 | 1 | N/A | 2 | 2 | 2 | 1 | 2 | 2 | 1 | 2 | 0 |
| Buitenweg et al., 2019^[4]^ | 2 | 2 | 2 | 1 | 2 | 2 | 1 | N/A | 2 | 2 | 1 | 1 | 2 | 2 | 2 | 2 | 0 |
| McGreevy, 2017^[5]^ | 1 | 2 | 2 | N/A | 2 | N/A | N/A | N/A | N/A | N/A | N/A | N/A | N/A | N/A | N/A | N/A | N/A |
| Johnson et al., 2016^[6]^ | 2 | 2 | 2 | 2 | 2 | 2 | 1 | N/A | 2 | 2 | 1 | 2 | 2 | 0 | 2 | 2 | 0 |
| Ross et al., 2022^[7]^ | 2 | 2 | 2 | 1 | 2 | 2 | 1 | N/A | 2 | 1 | 2 | 2 | 2 | 0 | 2 | 2 | 0 |
| Article | Item 1 | Item 2 | Item 3 | Item 4 | Item 5 | Item 6 | Item 7 | Item 8 | Item 9 | Item 10 | Item 11 | Item 12 | Item 13 | Item 14 | Item 15 | Item 16 | Item 17 |
| Ter Harmsel et al., 2023a^[8]^ | 2 | 2 | 2 | 2 | 2 | 2 | 2 | N/A | 2 | 1 | 2 | 1 | 2 | 1 | 2 | 2 | 2 |
| Ter Harmsel, et al., 2023b^[9]^ | 2 | 2 | 2 | 2 | 2 | 2 | 1 | N/A | 2 | 2 | 2 | 1 | 2 | 1 | 2 | 2 | 2 |
| Ter Harmsel, et al., 2021^[10]^ | 2 | 2 | 2 | 2 | 2 | 2 | 1 | N/A | 2 | 2 | 2 | 2 | 2 | 2 | 2 | 2 | 2 |
| Sugie, 2018^[11]^ | 2 | 2 | 2 | 2 | 2 | 2 | 1 | N/A | 1 | 2 | 1 | 2 | 2 | 2 | 2 | 2 | 0 |
| Langdon et al., 2022^[12]^ | 2 | 2 | 2 | 2 | 2 | 2 | 1 | N/A | 2 | 1 | 2 | 1 | 2 | 2 | 2 | 2 | 0 |
| Perdacher et al., 2022^[13]^ | 2 | 2 | 2 | 2 | 2 | 2 | 1 | N/A | 2 | 1 | 1 | 1 | 2 | 2 | 2 | 2 | 0 |
| Perdacher et al., 2024^[14]^ | 2 | 2 | 2 | 2 | 2 | 2 | 1 | 1 | 2 | 2 | 1 | 1 | 2 | 2 | 2 | 2 | 1 |
| Schuler et al., 2021^[15]^ | 2 | 2 | 2 | 2 | 2 | 2 | N/A | N/A | 1 | 2 | 1 | 2 | 2 | 2 | 1 | 2 | 0 |
| Article | Item 1 | Item 2 | Item 3 | Item 4 | Item 5 | Item 6 | Item 7 | Item 8 | Item 9 | Item 10 | Item 11 | Item 12 | Item 13 | Item 14 | Item 15 | Item 16 | Item 17 |
| Lau et al., 2022^[16]^ | 2 | 2 | 2 | 2 | 1 | 1 | 1 | N/A | 1 | 1 | 2 | 1 | 2 | 0 | 2 | 0 | 0 |
| Sugie & Augustine, 2020^[17]^ | 1 | 2 | 2 | 2 | 2 | 2 | 1 | N/A | 2 | 2 | 1 | 2 | 2 | 2 | 2 | 2 | 0 |
| Cox, 2016^[18]^ | 1 | 2 | 2 | 2 | 2 | 2 | 1 | N/A | 2 | 2 | 1 | 2 | 2 | 2 | 1 | 2 | 0 |
| Burraston et al., 2014^[19]^ | 2 | 2 | 2 | 2 | 2 | 2 | 1 | N/A | 2 | 1 | 2 | 2 | 2 | 2 | 2 | 2 | 0 |
| Burraston et al., 2012^[20]^ | 2 | 2 | 2 | 2 | 2 | 2 | 1 | N/A | 2 | 1 | 2 | 2 | 2 | 2 | 1 | 2 | 0 |
| Leijse et al., 2024^[21]^ | 2 | 2 | 2 | 2 | 2 | 2 | 1 | N/A | 2 | 1 | 1 | 2 | 2 | 2 | 2 | 2 | 0 |
| McCrady et al., 2025^[22]^ | 2 | 2 | 2 | 2 | 2 | 2 | 1 | N/A | 1 | 1 | 1 | 2 | 2 | 2 | 2 | 2 | 0 |
